# Medical Journals

**Published:** 1857-02

**Authors:** 


*Medical Journals.*

The *Medical Examiner* of Philadelphia and the *Louisville Review* have been united and merged in one large bi-monthly journal called the *North American Medico-Chirurgical Review;* and edited by S. D. Gross, M.D. Professor of Surgery in Jefferson Medical College, and T. G. Richardson, M.D. Professor of Anatomy in the Pennsylvania Medical College. The *Review* contains one hundred and sixty pages, printed in excellent style by J. B. Lippencott & Co. Philadelphia. The well-known ability and industry of its editors affords the strongest possible guarantee of its value and success.

The *Southern Journal of Medical and Physical Sciences*, which was suspended for a time, has re-appeared with renewed vigor. It is edited with ability, and we trust it may have a more *uniform* existence in future.

